# Genomic consequences of multiple speciation processes in a stick insect

**DOI:** 10.1098/rspb.2012.0813

**Published:** 2012-10-13

**Authors:** Patrik Nosil, Zach Gompert, Timothy E. Farkas, Aaron A. Comeault, Jeffrey L. Feder, C. Alex Buerkle, Thomas L. Parchman

**Affiliations:** 1 Department of Ecology and Evolutionary Biology, University of Colorado, Boulder, CO 80303, USA; 2 Department of Botany, University of Wyoming, Laramie, WY 82071, USA; 3 Department of Biology, Notre Dame University, South Bend, IN 11111, USA

**Keywords:** gene flow, genomic divergence, natural selection, reproductive isolation, *Timema*

## Abstract

Diverse geographical modes and mechanisms of speciation are known, and individual speciation genes have now been identified. Despite this progress, genome-wide outcomes of different evolutionary processes during speciation are less understood. Here, we integrate ecological and spatial information, mating trials, transplantation data and analysis of 86 130 single nucleotide polymorphisms (SNPs) in eight populations (28 pairwise comparisons) of *Timema cristinae* stick insects to test the effects of different factors on genomic divergence in a system undergoing ecological speciation. We find patterns consistent with effects of numerous factors, including geographical distance, gene flow, divergence in host plant use and climate, and selection against maladaptive hybridization (i.e. reinforcement). For example, the number of highly differentiated ‘outlier loci’, allele-frequency clines and the overall distribution of genomic differentiation were recognizably affected by these factors. Although host use has strong effects on phenotypic divergence and reproductive isolation, its effects on genomic divergence were subtler and other factors had pronounced effects. The results demonstrate how genomic data can provide new insights into speciation and how genomic divergence can be complex, yet predictable. Future work could adopt experimental, mapping and functional approaches to directly test which genetic regions are affected by selection and determine their physical location in the genome.

## 1. Introduction

New species can form under conditions of geographical overlap or separation [1,2], and through the action of different evolutionary processes. Thus, geographical mode and mechanism of speciation are a focus of speciation research [3,4]. Mayr and others [5,6] argued that geographical isolation promotes speciation by reducing homogenizing gene flow, and indeed numerous examples of allopatric speciation exist [3,7]. Additionally, the geographical arrangement of populations can have consequences beyond affecting gene flow. For example, increased geographical separation might expose populations to greater divergent selection [3,7]. This could occur if populations that are farther apart are exposed to stronger divergent selection along environmental gradients or a greater diversity of selection pressures. In such instances, selection might cause ‘ecological speciation’ [8–11]. In other instances, geographical contact between taxa might actually promote, rather than constrain, speciation. One possibility is ‘reinforcement’, in which selection against unfit hybrids in geographical regions of contact drives the evolution of premating isolation [12–14]. Thus, a variety of speciation mechanisms are known and individual genes implicated in adaptation and speciation have been described [15–18].

In contrast to the classical study of mode and mechanism of speciation, the emerging field of speciation genomics is in its infancy [19]. The field has usefully revealed that divergence is highly heterogeneous across the genome, but is still in a phase where explicit and non-overlapping predictions associated with many hypotheses have yet to emerge [20–22]. Thus, genomic data from populations that are variably isolated on ecological and spatial scales have the potential to advance our conceptual understanding of the interaction and relative importance of different processes in shaping genomic divergence. Our goal here is to take advantage of recent advances in DNA sequencing and an ecologically characterized system that allows replicated comparisons across populations to conduct an analysis of the genomic consequences of mode and mechanism of speciation.

Although non-overlapping predictions concerning the specific causes of patterns of genomic divergence are still being developed, a number of *a priori* predictions can be made for the manner in which divergence will be affected by particular processes [19]. For example, although many processes affect the distribution of genomic differentiation across loci, gene flow could do so in predictable ways. Speciation with gene flow, either primary or following secondary contact, might be characterized by divergence in only a few regions that harbour genes under strong divergent selection and those causing reproductive isolation [23,24], while the rest of the genome is homogenized by gene flow. This is predicted to generate an ‘L-shaped’ frequency distribution of genetic differentiation across loci (i.e. most loci have low *F*_ST_ values). In contrast, allopatric speciation might be characterized by divergence across more of the genome, leading to a different distribution of genomic differentiation than observed with gene flow [19]. Additionally, processes other than gene flow might leave predictable genomic patterns. For example, gene regions involved in ecological speciation should be strongly differentiated between ecologically divergent, but not ecologically similar, population pairs. Likewise, reinforcement should result in some loci being strongly differentiated only between adjacent, hybridizing population pairs.

Our goal here is to assess these predictions and their applicability in population comparisons of *Timema cristinae* stick insects. We collected genomic data from a mosaic of populations where past work clearly indicates that numerous factors, such as divergent host adaptation, geographical separation, gene flow and reinforcement all affect speciation [25]. The genotyping-by-sequencing approach was to use restriction enzymes to cut up the genome into DNA fragments that are distributed across the genome, sequence tens of millions of these fragments on the Illumina next-generation sequencing (NGS) platform, align the fragments to discover genetic variation (facilitated by specimens being individually barcoded), and then conduct population genetic analyses on the resulting 86 130 single nucleotide polymorphisms (SNPs). This approach is thus aimed at surveying genome-wide patterns of genetic differentiation across the approximately 1.3 gigabase genome of *T. cristinae*, rather than focusing in on specific genes that causally affect adaptation and speciation. Our Bayesian analyses incorporate genotype uncertainty, and thus account for the uneven coverage among gene regions and individuals inherent in NGS data.

The results demonstrate interplay between different speciation processes, each of which is associated with different patterns of genomic divergence. Surprisingly, we find that the effects of host use on genomic divergence are subtle, despite the strong effects of host use on ecological speciation, and that factors other than host use have pronounced effects on genomic divergence. The results highlight how genomic data have great potential for advancing understanding of speciation.

## 2. Study system

*Timema cristinae* is found feeding on two strikingly different host plant species in southern California (*Adenostoma fasciculatum*: Rosaceae and *Ceanothus spinosus*: Rhamnaceae: electronic supplementary materials, for details). There is strong evidence that divergent host adaptation has contributed to phenotypic divergence (e.g. in morphological traits related to crypsis) and reproductive isolation, and is thus causing ecological speciation [26]. For example, pairs of populations feeding on different host species (host ‘ecotypes’) exhibit stronger sexual isolation than pairs of populations feeding on the same host species.

Furthermore, populations can be geographically separated from one another (i.e. by regions containing unsuitable hosts) or in direct contact (‘geographically adjacent’ hereafter). Past work indicates that adjacent population pairs experience more gene flow than separated pairs, but that mating isolation between adjacent pairs has nonetheless been promoted by reinforcement [25–28]. Hybrids between the ecotypes are intrinsically viable and fertile, but are often intermediate for morphological characters subject to divergent selection between hosts, and thus suffer reduced fitness on each host [29,30]. This extrinsic postmating isolation creates the opportunity for reinforcement. As predicted by reinforcement, mating isolation is consistently stronger between adjacent populations than between separated ones [26].

In contrast to mating isolation, divergence of other characters is constrained by gene flow. Morphological traits such as size, shape and coloration are less divergent between geographically adjacent than geographically separated populations [25,27]. For example, the presence versus absence of a heritable dorsal stripe is subject to strong divergent selection, but its degree of population differentiation is negatively related to levels of gene flow [25,27,29]. Similar patterns occur for extrinsic reproductive isolation owing to ecological selection against between-host migrants and hybrids [25]. Thus, while gene flow allows for the evolution of mating isolation via reinforcement, it constrains adaptive divergence in morphology and extrinsic reproductive isolation. *Timema cristinae* is therefore ideal for analysing the genomic consequences of multiple evolutionary processes.

## 3. Material and methods

### (a) Mating trials and morph frequency data

We quantified sexual isolation and morphological divergence for the specific pairs of populations examined for genomic divergence (see electronic supplementary material, tables S1 and S2 for population information). Previously published protocols were used to conduct no-choice mating trials [26,31] during spring 2009. In brief, one male and one female were placed in a 10 cm Petri dish and at the end of 1 h we scored whether or not copulation had occurred. The individuals used were field-collected virgins. Past work has demonstrated mating isolation to be unaffected by rearing environment and thus likely highly heritable [26]. Individuals were selected randomly from each population, such that mating trials were conducted using natural morph frequencies. The degree of mating isolation was calculated as 1 – (% between-population mating/% within-population mating). A *t*-test was used to test whether the degree of mating isolation differed between separated versus adjacent population pairs.

**Figure 1.**
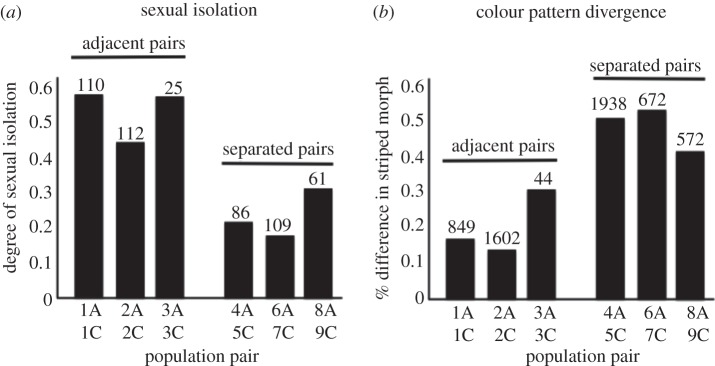
Evolutionary divergence between geographically adjacent versus geographically separated population pairs of *Timema cristinae*. Codes on the *x*-axis label the population pairs (A refers to use of *Adenostoma* as a host and C to use of *Ceanothus*)*.* (*a*) Sexual isolation is stronger between adjacent versus separated populations (*p* < 0.01), indicative of reinforcement. Numbers above bars represent number of mating pairs tested. (*b*) Divergence in cryptic coloration is weaker between adjacent versus separated populations (*p* < 0.05), indicative of homogenizing gene flow. Numbers above bars represent number of individuals scored for the presence versus absence of a dorsal stripe.

Most of the morph frequency data analysed here stem from previously published datasets [32,33]. The exception was data for populations MR1A and MR1C, where individuals were collected with sweep nets in March 2010 and scored for the presence or absence of a stripe. Past studies have shown morph frequencies to be highly divergent between hosts [25,29]. These spatial differences in host use far exceed temporal fluctuations, at least on the scale of decades [33]. An independent sample *t*-test was used to test whether the degree of divergence between populations in the proportion of striped individuals (% striped on *Adenostoma* − % striped on *Ceanothus*) differed between geographically separated versus geographically adjacent population pairs. Labels for populations for which genomic data were not collected are as follows: R23A is 6A, R6C is 7C, LOGA is 8A and PC is 9C (figure 1).

### (b) Sequencing protocols

To characterize genomic divergence, we generated genome-wide sequence data from eight populations of *T. cristinae* via sequencing of reduced complexity genomic libraries (*n* = 161 individuals; electronic supplementary material, tables S1 and S2). DNA was extracted from the legs of *T. cristinae* using Qiagen DNeasy kits. We used genomic enrichment prior to high-throughput sequencing involving a restriction enzyme digestion step followed by PCR amplification to produce a pool of fragments for sequencing [34,35]. Illumina sequencing adaptors and individual barcodes were ligated onto the ends of each fragment allowing highly multiplexed sequencing. Details concerning adaptor-ligation, subsequent fragment amplification via PCR, and single-end sequencing starting from the *Eco*RI adaptor on two lanes of an Illumina GAIIx genetic analyser are described in the electronic supplementary material.

### (c) Assembly

An average of 284 896 reads were generated per barcoded individual. We executed a de novo assembly on a set of 20 million reads using SeqMan Ngen v. 3.0 (DNAstar, Inc.). The de novo assembly placed 10 852 133 reads into 293 614 contigs containing a minimum of eight reads for an average coverage depth per contig across all individuals of 36×. We used the consensus sequences from the contigs constructed during de novo assembly to construct an artificial reference, and assembled the complete set of 46 153 271 reads onto this reference using a template-guided assembly. This placed 34 357 655 reads onto the reference sequences, and resulted in an average coverage depth of 125× per genetic region across all individuals (0.77× per individual; the electronic supplementary material, table S1 for coverage per population). After calling variants using bcftools in samtools and further quality trimming, 86 130 variant sites (i.e. SNPs) spanning 57 969 contigs were used in subsequent analyses. Details are in the electronic supplementary material.

### (d) Population differentiation and linkage disequilibrium

We used a Bayesian model to estimate population allele frequencies for each SNP based on the observed sequence data [36,37]. The allele frequency model incorporates uncertainty in genotypic state that results from low coverage and missing data, which is a common occurrence with high-throughput sequencing. Genotypic states and allele frequencies were therefore treated as model parameters and estimated using Markov chain Monte Carlo (MCMC) following [37]. Thus, all 86 130 SNPs were represented in each of the 28 pairwise population comparisons. We used 20 000 MCMC iterations that were thinned to every fourth sample to estimate posterior probabilities for these parameters. We then used principal component analysis (PCA) to summarize genetic variation among populations. We used the posterior probabilities of each of two genotypic states for each locus as variables for PCA. We performed PCA in R using the *prcomp* function (R Development Team).

We used a hierarchical Bayesian implementation of the F-model to quantify genetic differentiation among populations (electronic supplementary material for details). Statistical outlier loci (i.e. loci with exceptionally high levels of population divergence) between individual population pairs were identified based on estimates of **α*_i_* relative to the estimated genome-wide distribution of **α** (i.e. logit(*F*_ST_)) as described in earlier studies by Gompert and co-workers [36,37]. The estimated genome-wide distribution of **α** is ∫∫Normal(**μ*′, *τ*′*)d**μ**d**τ**, where **μ*′* and **τ*′* are the posterior probability distributions of **μ** and **τ**. Specifically, **α** is a vector with one element for each locus. Each element of **α** is the logit *F*_ST_ for a locus, **μ** is the mean or genome level logit *F*_ST_, **τ** describes variation in *F*_ST_ among loci, and is equal to the precision (one divided by the variance) of locus-specific logit *F*_ST_.

We called loci statistical outliers if the posterior point estimate of **α*_i_* was not contained in the interval *q_N_*, where *q_N_* is the interval bounded by the 95th and (1*–*95th) quantiles of the genome-wide distribution of **α**. We tested for associations between allele frequencies and bioclimatic variables using methods described in the electronic supplementary material.

We estimated Burrow's composite measure of Hardy–Weinberg and linkage disequilibrium (LD; *Δ*) for each pair of variable sites [38]. This measure does not assume Hardy–Weinberg equilibrium or require phased data, but instead provides a joint metric of intralocus and interlocus disequilibria based solely on genotype frequencies [38]. These estimates are constrained by allele frequencies at each locus, and the maximum value is lower for loci with lower minor allele frequencies. Notably, *Δ* is a population-level measure, and per population coverage was relatively high even when per individual coverage was lower.

### (e) Approximate Bayesian computation of gene flow

We used approximate Bayesian computation (ABC) to model historical divergence and gene flow among the 28 population pairs. Specifically, we compared two alternative models for each pair of populations: (i) divergence without gene flow, and (ii) divergence with gene flow. See the electronic supplementary material for details.

### (f) Matrix correlation analyses

We used simple and partial Mantel randomization tests to compare various matrices with one another while accounting for potential non-independence of matrix elements, as described in the electronic supplementary material [39]. For example, matrices of geographical and genetic distance and matrices of geographical distance and number of outliers were compared in this fashion.

### (g) Transplantation data

Insects were transplanted to one general area (approx. 1 km^2^ area surrounding N34 30.958 W119 48.050) from two populations that varied in their distance from the transplant site. The individuals used in this experiment were from populations that are phenotypically variable for traits involved in host adaptation (owing to gene flow) and are thus expected to survive, on average, equally well on each host species. Thus, these experiments examine the effects of geographical distance, rather than host, on survival. The first transplant involved the population R12C that is located approximately 25 km from the transplantation site. This corresponds to the maximum distance in our genomic sampling and therefore represents a ‘distant’ transplant. The second transplant involved the population FHA (N34 31.089 W119 48.166) located about 1 km from the transplant site, representing a ‘near’ transplant. We used ANOVA to test whether the proportion of survivors (i.e. % recaptured) in each transplant was dependent on the transplant (fixed factor), host (fixed factor) and block (random factor). We tested a full model and a reduced one containing only main effects.

## 4. Results and discussion

### (a) Reinforcement, morphological divergence and gene flow

Past work clearly demonstrated reinforcement of mating preference in multiple adjacent population pairs of *T. cristinae* [25,26]. New mating data (*n* = 503 trials) demonstrated that the specific adjacent population pairs examined here for genomic divergence exhibited twice the mating isolation of separated pairs (mean isolation index = 0.54 versus 0.24, *t* = 5.02, d.f. = 4, *p* = 0.007, *t*-test; figure 1). In contrast, the difference in morph frequency for the adjacent pairs examined here was less than half that observed for separated pairs (mean difference between hosts in % striped individuals = 35% versus 84%, *t* = 4.61, d.f. = 4, *p* = 0.010, *n* = 5677; figure 1). These data confirm the dual effects of geographical contact for the adjacent populations examined here.

### (b) Genomic divergence

The genomic data yielded 86 130 SNPs. We used Bayesian models to estimate allele frequencies (electronic supplementary material, figure S1), genotype probabilities and genome-wide differentiation between all possible pairwise comparisons between the study populations (*n* = 28). The results revealed highly variable genetic differentiation between populations. Specifically, average differentiation was relatively strong (mean *F*_ST_ = 0.111), but ranged among population pairs from very low (minimum *F*_ST_ = 0.007) to very high (maximum *F*_ST_ = 0.306). PCA based on genotypic state posterior probabilities revealed genetic clustering according to the geographical distances between populations (electronic supplementary material, figure S2; i.e. isolation-by-distance, *r* = 0.96, *p* = 0.001, Mantel test).

Information about the genomic distribution of the SNP loci will require future linkage mapping studies or a whole genome reference sequence. However, estimates of Burrow's composite measure of Hardy–Weinberg and linkage disequilibrium (*Δ*) within populations were very low, indicating these SNPs were largely statistically independent from one another and did not generally exhibit pronounced departures from Hardy–Weinberg equilibrium (e.g. mean *Δ* across the approx. 1.9 billion locus pairs was on the order of 0.003; electronic supplementary material, table S3 for full results) [38]. SNPs within the same contig had somewhat elevated LD relative to those from different contigs (in the order of double), but still had low LD overall (electronic supplementary material, table S3). The proportion of all possible pairs of SNPs on different contigs was greater than 0.999.

Investigations of genomic divergence often focus on the identification and enumeration of loci with unusually high levels of population differentiation. We used a Bayesian method developed for next-generation sequence data to identify such ‘outlier loci’ that are statistically diverged beyond background null expectations [36,37]. Outlier loci are thought to often reside in regions affected by divergent selection and indeed past theoretical work has shown outlier loci are enriched for such regions [19]. However, a number of processes other than divergent selection, such as low recombination rate and genetic drift, can also promote accentuated genetic divergence. Thus, we use the number of outlier loci as a summary statistic for the number of exceptionally differentiated SNPs, without claiming all outliers are affected by selection.

**Figure 2.**
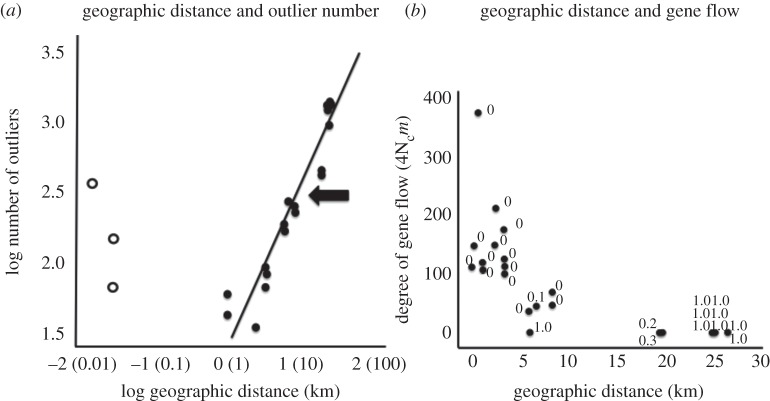
Geographical distance, outlier number and gene flow. (*a*) The relationship between the number of outliers and geographical distance (both on log_10_ scale), for geographically separated and geographically adjacent population pairs (filled and unfilled circles, respectively). Both the effects of geographical distance itself, and of geographical arrangement (separated versus adjacent) were statistically significant. The thick arrow labels the point at which zero gene flow between population pairs was inferred (see *b* and electronic supplementary material, table S2). (*b*) Estimates of gene flow fall to zero before the maximum separation distance between population pairs examined. The numbers beside each data point refer to the posterior probability of a model with zero gene flow.

A total of 15 207 loci were categorized as statistical outliers. Absolute levels of *Δ* for outlier loci were low, and not markedly different from that of all loci (e.g. mean difference = 0.0008, *t*_7_ = 1.37, *p* = 0.21, paired *t*-test; electronic supplementary material, table S3). Thus, outliers appear to largely represent independent markers of population divergence and not tightly linked blocks of loci, but confirmation of this awaits mapping studies. The number of outlier loci varied drastically among population pairs (range = 34–1364, mean = 543), providing the requisite variation to test which factors affect this variability. Two factors clearly and significantly did so (figure 2; electronic supplementary material, table S4 for statistics). First, the number of outlier loci increased with geographical distance between populations. Second, the geographical arrangement of populations was related to the number of outliers. Specifically, adjacent pairs exhibited an excess of outliers relative to what would be predicted on the basis of variation among geographically separated pairs. The resulting pattern was a curvilinear (i.e. ‘U-shaped’) relationship between the number of outliers and geographical distance between populations. In contrast to the other factors, divergence in host plant use was not related to the number of outliers (503 versus 597 on average for different-host versus same-host pairs, respectively, all *p* > 0.40 in Mantel tests).

These patterns raise four fundamental questions: (i) does host adaptation recognizably affect genomic differentiation? (ii) Is the enrichment of outliers between adjacent population pairs a consequence of reinforcement? (iii) Why do the number of outliers increase with increased geographic separation? (iv) Are the genomic effects of these factors restricted to outlier loci? We address each question in turn.

### (c) Host use and genomic divergence

**Figure 3.**
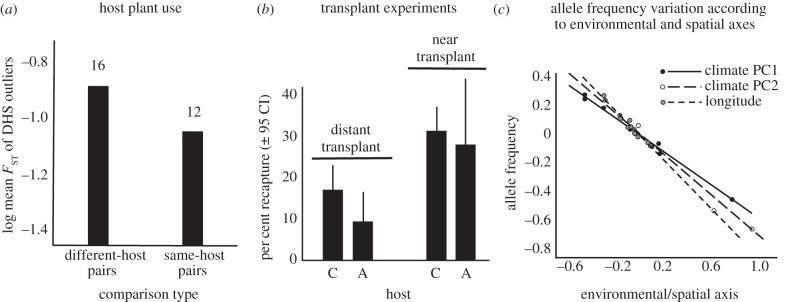
The effect of factors other than gene flow on evolution. (*a*) Differentiation (*F*_ST_) of the 11 outliers that were specific to different-host pairs and that arose in at least three pairs of populations using different hosts (DHS outliers). Significant overall effects of divergence in host use on *F*_ST_ were observed across the 28 population pairs examined (both *p* < 0.05, partial Mantel tests controlling for median *F*_ST_ on raw and log-transformed data, respectively). Numbers above bars denote numbers of population pairs. (*b*) Recapture probability, a proxy for survival, in the same general area was lower when individuals were transplanted from a distant versus a near source population. (*c*) Variation in allele frequencies as a function of environmental and spatial axes. Data are shown for an outlier locus (SNP 47457; electronic supplementary material, table S6 for statistics and results from other loci). Partial plots are shown from a multiple regression analysis that included climatic PC1 (indicative of temperature and precipitation), climatic PC2 (indicative of climatic variability), and longitude. Values on *x*-axis are standardized *z*-scores and on the *y*-axis are standardized residuals from the regression.

We examined the characteristics of outlier loci by enumerating the number and types of pairwise comparisons in which they were outliers (the electronic supplementary material, table S5). Although the number of outlier loci was not related to divergence in host use, some loci were associated with divergent host use. For example, 529 loci were observed as outliers in different-host pairs but never exhibited outlier status in any same-host pair (with 59 being outliers in multiple different-host comparisons). The outliers specific to different-host pairs are likely to reside in regions affected by host-specific selection. Their relatively low numbers could be due to the small fraction of the overall genome sequenced and due to gene flow preventing strong differentiation at loci subject to weak host-specific selection. Consistent with the latter hypothesis, mean *F*_ST_ for outliers specific to different-host pairs across all 28 population pairs (i.e. not just those in which they were outliers) was significantly greater for different-host than same-host-pairs (figure 3). Thus, host adaptation left a clear but subtle pattern in the genome.

### (d) Reinforcement and genomic divergence

As predicted by reinforcement, of the hundreds of outliers found between geographically adjacent pairs, almost all (493 of 554 = 89%) were restricted to being outliers between adjacent population pairs and were thus never outliers in the 25 comparisons between geographically separated pairs. The excess of outliers between adjacent pairs cannot be explained by accentuated climatic differences between such pairs, because adjacent pairs are the least climatically divergent (electronic supplementary material, table S2).

### (e) Causes of association between geographical distance and outlier number

The observed increase in genomic differentiation with increased geographical distance between populations could be explained by a number of processes, including stronger divergent selection, weaker gene flow, demographic variability and genetic drift, mutation rate variation or a combination of these factors [3]. To test the contributions of these various processes, we first examined the spatial scale of gene flow, which, owing to winglessness, is small in *T. cristinae*. For example, mark–recapture data estimated that the average per-generation dispersal distance was only 12 m [40] and manipulating adjacent patches to be separated by 36 m reduced gene flow to the extent that increased adaptive divergence occurred after only a single generation [28]. If the absence of gene flow occurs at a spatial scale smaller than that of our study, it would indicate that any observed increases in the number of outliers beyond that scale of zero gene flow were due to factors other than further reduced gene flow.

We used ABC to estimate gene flow from the genomic data under a model of isolation with migration. The results revealed that gene flow decreased sharply with increased distance between populations (*r* = −0.75, −0.71, both *p* < 0.01, Mantel tests on raw and log-transformed data, respectively). Zero gene flow was observed at a scale below the maximum 25 km distance examined (figure 2 and the electronic supplementary material, table S2). For example, estimates of migration (4*N*_e_*m*) fall to zero for populations separated by more than 10 km. Likewise, posterior probabilities for a model of zero gene flow increase abruptly from near zero to one at around 15 km of separation. Thus, factors other than reduced gene flow likely affect the number of outliers observed.

For example, transplantation data suggest the possibility of stronger selection between more distant populations. An experiment definitively testing the ‘stronger selection’ hypothesis by transplanting insects from a single population various distances has yet to be conducted. Here, we implemented two transplants in the field, where individuals were moved to the same general area from source populations that were either distant (25 km) or near (1 km) the transplant area. When individuals were transplanted from a distant source population, their survival 8 days later was significantly lower compared with individuals transplanted a short distance (main effect of distance, full model, *F*_1,4_ = 14.00, *p* = 0.02, reduced model, *F*_1,13_ = 18.75, *p* = 0.001, all other terms, *p* > 0.10). This pattern is consistent with stronger divergent selection between distant than nearby populations, but further data are required to confirm this (figure 3).

Finally, we tested for clinal variation in allele frequencies along environmental and spatial axes, as predicted if selection varies spatially. Specifically, we examined relationships between allele frequencies within populations and three continuous variables (two multivariate climatic variables and longitude; figure 3 and electronic supplementary material, table S6). We did so for the 16 most repeated outlier loci and for 16 randomly chosen loci (i.e. 48 clines tested for each class of locus). We detected relationships between allele frequencies and both environmental and spatial axes. Significant relationships were more common for outlier than random loci (30 versus 4, respectively) and were sometimes overlaid on host effects (i.e. parallel clines between hosts with differing *y*-intercepts). Thus, allele frequencies were not only affected by host plant use and geography, but also by climate. In sum, reduced gene flow likely contributes to an increased number of outliers with increasing geographical separation, but other factors such as increased selection or drift are likely involved as well, particularly at distances beyond 10–15 km.

### (f) Effects of geographical distance on the overall distribution of differentiation

**Figure 4.**
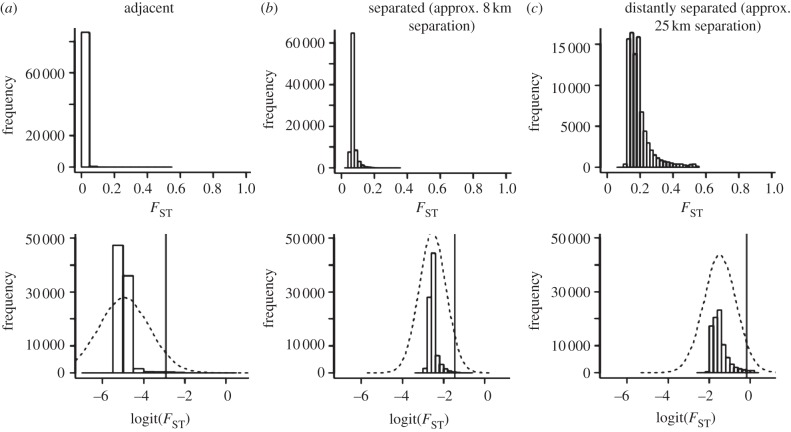
The distribution of *F*_ST_ values under different degrees of geographical separation. The top panel shows the distribution of *F*_ST_ values across individual loci for three different pairwise comparisons: (*a*) 1A × 1C. (*b*) 2A × 5C. (*c*) 2A × 1C. The bottom panel presents a bar plot of the distribution of point estimates for logit(*F*_ST_) across the genome for these same comparisons. The dashed black line is the genome-wide distribution of logit(*F*_ST_)(i.e. the Gaussian normal hierarchical prior for locus-specific logit(*F*_ST_)). The vertical line in each pane denotes the 95th quantile of the genome-wide distribution, which was used to delimit high *F*_ST_ outliers. The *F*_ST_ distribution tended to be the most ‘L-shaped’ for geographically adjacent pairs and became less ‘L-shaped’ with increasing geographical separation of populations. See electronic supplementary material for statistics involving all 28 pairwise comparisons.

The effects of geography were not restricted to outlier loci. The overall distribution of differentiation across the genome was also affected by geographical distance, as expected under population genetic models that predict both increased mean and variance of *F*_ST_ with reduced gene flow [41]. In particular, the shape of the *F*_ST_ distribution was more strongly ‘L-shaped’ for adjacent pairs than geographically separated pairs (figure 4). As a result, the *F*_ST_ distribution was significantly more skewed for adjacent relative to separated pairs (mean skewness values = 10.64 versus 3.32, all *p* < 0.005; electronic supplementary material, table S4), with less density in the centre and a more pronounced tail of extreme values (mean kurtosis values = 376 versus 19, all *p* < 0.05). Adjacent pairs, relative to separated pairs, exhibited significantly lower average *F*_ST_ values (0.02 versus 0.12, all *p* < 0.001) and less overall variance in *F*_ST_ among loci (0.001 versus 0.002, all *p* < 0.05), owing to adjacent pairs exhibiting many fewer loci with moderate *F*_ST_ values. Even among separated pairs, such patterns often became more accentuated as geographical distance between populations increased (electronic supplementary material, table S4). These results demonstrate widespread effects of geography on genomic divergence. Indeed, it could be that geographical separation in widespread species is similar to speciation in a ring, where gradual reductions in gene flow with distance facilitate speciation [3,42].

## 5. Conclusions

Our results have implications for understanding speciation. The ecotypes of *T. cristinae* are an example of the process of ecological speciation via host adaptation [9]. The findings presented here indicate that speciation in this system involves much more than just strong reproductive isolation evolving as an incidental by-product of divergent host use. At a minimum, the effects of host-related selection on speciation are mediated by the degree to which geographical isolation reduces homogenizing gene flow. At the other extreme, multifarious divergent selection on traits unrelated to host use could be important for generating widespread genomic divergence, and is increasingly expressed with greater geographical separation. Nonetheless, divergence in host use is critical owing to its role in ecologically based reinforcement. This interplay of factors results in a geographical mosaic of phenotypic and genomic evolution [43]. For example, with respect to the number of outlier loci, the relative importance of being separated by 10 km, the point of near zero gene flow, is roughly equivalent to that of reinforcement between adjacent populations (approx. 500 outlier loci in both cases), and both of these effects are overlaid on that of host use.

Our results highlight how the speciation process can have complex, but predictable, effects on genomic divergence, that reflect the consequences of geographical separation, gene flow, multifarious divergent adaptation, reinforcement and other evolutionary processes. Future work using experimental, mapping and functional genomics approaches to directly test which genetic regions are affected by selection, and to determine their physical distribution across the genome, will likely yield further insight into the speciation process.
